# How Do Visual and Parietal Cortex Contribute to Visual Short-Term Memory?^1^,^2^

**DOI:** 10.1523/ENEURO.0041-16.2016

**Published:** 2016-05-03

**Authors:** Edward F. Ester, Rosanne L. Rademaker, Thomas C. Sprague

**Affiliations:** 1 Department of Psychology, University of California, San Diego, La Jolla, California 92093; 2 Neurosciences Graduate Program, University of California, San Diego, La Jolla, California 92093; 3 Department of Psychology, New York University, New York, New York 10003

**Keywords:** fMRI, multivoxel pattern analysis, parietal cortex, short-term memory, visual cortex

Visual short-term memory (VSTM) enables the representation and manipulation of information no longer present in the sensorium. VSTM storage has long been associated with sustained increases in univariate activity (eg, averaged single-neuron spike counts or fMRI activation levels) across a broad network of frontal and parietal cortical areas (for review, see D’Esposito and Postle, 2015). More recently, several research groups have used multivariate analytical techniques to “decode” or infer the identity of a remembered visual stimulus from multivariate fMRI responses measured in human visual cortical areas (eg, V1–V4) during the delay period of a VSTM task, even though these areas typically do not show sustained increases in activity during VSTM storage (Harrison and Tong, 2009; Serences et al., 2009; Riggall and Postle, 2012; Emrich et al., 2013; van Bergen et al., 2015). However, virtually all of these studies have used simple designs that require participants to remember information over a blank delay period. In many real world scenarios, information must be stored despite a constant barrage of dynamic and unpredictable sensory input. How does the brain accomplish this goal?

A recent human neuroimaging paper by Bettencourt and Xu (2016) attempted to answer precisely this question. In their Experiment 1, participants were shown a sequence of two tilted gratings and retroactively cued to remember the orientation of either the first or the second grating. Following a blank delay period, participants judged whether the orientation of a probe grating was tilted slightly clockwise or anticlockwise of the remembered orientation. During the first half of experimental blocks, participants remembered the cued orientation over a blank delay (no-distractor blocks). During the second half of blocks, the delay period was filled with a sequence of task-irrelevant images (photographs of faces or gazebos; distractor blocks). Using fMRI and a multivariate pattern classification algorithm, the authors attempted to decode the orientation of the remembered grating from delay-period activation patterns measured in visual and parietal cortex as a function of distractor presence. In particular, the authors focused on two regions-of-interest (ROIs). The first encompassed retinotopically organized visual areas V1–V4, which typically support robust decoding of remembered stimuli (Harrison and Tong, 2009; Serences et al., 2009). The second encompassed portions of the superior intraparietal sulcus (sIPS), where overall activation levels have been shown to track the amount of task-relevant information that must be enumerated (Nieder et al., 2006; Harvey et al., 2013), tracked (Drew and Vogel, 2008), attended (Mitchell and Cusack, 2008), or stored in VSTM (Todd and Marois, 2004; Xu and Chun, 2006).

On the assumption that areas V1–V4 would be recruited during sensory processing of the distractor photographs, Bettencourt and Xu (2016) reasoned that stimulus-specific delay period activation patterns in these regions would be particularly susceptible to interference. By contrast, insofar as sIPS is less involved in sensory processing, stimulus-specific delay period activation patterns in measured this region should be robust to interference. Thus, Bettencourt and Xu (2016) hypothesized that decoding performance in V1–V4 would be reduced during distractor blocks relative to no-distractor blocks, whereas decoding performance in sIPS would be unaffected by distractor presence. This is precisely what was found: during no-distractor blocks, both V1–V4 and sIPS supported above-chance decoding of the cued orientation, replicating earlier findings (Ester et al., 2009; Harrison and Tong, 2009; Serences et al., 2009; Christophel et al., 2012; Emrich et al., 2013; van Bergen et al., 2015). During distractor blocks, decoding performance in sIPS remained well above chance levels (and statistically indistinguishable from decoding performance on no-distractor blocks), whereas decoding performance in V1–V4 was reduced to chance. Additionally, participants’ behavioral performance was statistically equivalent during distractor and no-distractor blocks, suggesting that the loss of stimulus-specific information in V1–V4 during distractor-present blocks had a negligible effect on memory performance. The authors interpreted these findings as evidence that sIPS, and not V1–V4, has a privileged role in mediating VSTM storage, particularly when distracting visual information is present.

This conclusion is problematic, because it rests on the assumption that if a particular region contributes VSTM, then activation patterns measured in that ROI during distractor blocks should support above-chance decoding of a remembered stimulus. However, chance-level decoding need not imply that a particular region does not contribute to VSTM (or any other related function). Indeed, one alternative possibility is that V1–V4 encode stimulus-specific representations during distractor blocks, but at a level of anatomical or physiological granularity that is inaccessible to the multivoxel decoding approaches used in this study (for a recent example of information existing at the level of single neurons, but not multivoxel activation patterns see Dubois et al., 2015). Thus, the results of this experiment provide only indirect support for the conclusion that sIPS has a privileged role in mediating VSTM storage.

In a subsequent experiment (Experiment 3), Bettencourt and Xu (2016) attempted to replicate these findings while mixing the distractor and no-distractor conditions within the same block of trials. If distractors invariably disrupt stimulus-specific VSTM representations in V1–V4, then the results of this experiment should be identical to their first experiment: both V1–V4 and sIPS should support above-chance decoding during no-distractor trials, but only sIPS should support above-chance decoding during distractor-present trials. However, in this version of the task *both* V1–V4 and sIPS supported above-chance decoding of the cued orientation during distractor trials. Bettencourt and Xu (2016) suggested that these results could reflect a strategic choice: if distractors selectively interfere with VSTM representations in V1–V4, then participants may choose to “disengage” these regions when they are certain that distractors will be present. This would explain why decoding performance in Experiment 1, where distractor and no-distractor trials were blocked, fell to chance levels when distractors were present. This seems unlikely for several reasons. First, recall that in Experiment 1 participants’ memory performance was equivalent during distractor and no-distractor blocks, even though decoding performance in V1–V4 fell to chance levels when distractors were present. Thus, activation patterns in V1–V4 made no discernable contribution to overall memory performance. It is therefore unclear why participants would choose to engage these regions under any circumstance (assuming this is indeed a strategic choice), particularly if doing so is metabolically costly. Second, as Bettencourt and Xu (2016) note, most real-world scenarios require VSTM representations to be maintained despite a constant barrage of dynamic and unpredictable sensory input. Thus, the results of Experiment 1, when distractor presence was entirely predictable, may reflect a unique (and artificial) set of circumstances rather than a general property of the neural systems supporting VSTM.

If V1–V4 and/or sIPS contribute to VSTM performance, then delay period activation patterns in these regions should correlate with participants’ memory performance (Emrich et al., 2013; Ester et al., 2013; van Bergen et al., 2015). In Experiment 4, Bettencourt and Xu (2016) tested this possibility by comparing activation patterns measured with fMRI with memory performance measured outside of the scanner. In both tasks, participants remembered the orientation of a single (masked) grating over a blank delay period. On each trial, the to-be-remembered grating was assigned one of six possible orientations (10°, 40°, 70°, 100°, 130°, or 160°). In the scanner, participants judged whether the orientation of a probe grating presented at the end of the trial was tilted clockwise or anticlockwise of the remembered orientation (as in Experiments 1 and 3). During behavioral testing, participants reported whether the remembered grating matched the orientation of a subsequent probe. During mismatch trials, the probe orientation was tilted ±30°, ±60°, or 90° relative to the remembered orientation. Bettencourt and Xu (2016) reasoned that representations that are more similar (eg, ±30° apart) should take longer to discriminate and should be harder to decode than representations that are less similar to one another (eg, ±90° apart). Consequently, decoding performance for similar pairs of remembered orientations should be inversely correlated with behavioral response latencies for the same pairs. That is, decoding accuracies should be low, and response latencies should be high, for similar relative to dissimilar pairs of orientations. Indeed, pairwise decoding accuracies were negatively correlated with pairwise response latencies in both V1–V4 (*r* = −0.70) and sIPS (*r* = −0.59). This result suggests that both V1–V4 and sIPS contribute to VSTM storage. Instead, Bettencourt and Xu (2016) argued that whereas the correlation between behavioral response times and neural activation pattern discriminability in sIPS likely reflects VSTM storage, the correlation between the same variables in V1–V4 likely reflects lingering sensory processing of the to-be-remembered stimulus. However, both correlations were generated using activation patterns measured during the same delay period interval, and both correlations remained strong when activation patterns measured during the early part of the memory delay (ie, those most likely to include contributions from lingering sensory responses) were omitted from the analysis. In light of these observations, it seems quite unlikely that the correlation reported in V1–V4 reflects sensory processing, whereas the correlation reported for sIPS reflects VSTM storage. Finally, note that no distractors were presented in this experiment. It would be interesting to know whether correlations between memory performance and activation patterns in V1–V4 and sIPS are modulated by distractor presence.

In our view, the data reported by Bettencourt and Xu (2016) provide only modest support for the assertion that sIPS has a central or privileged role in mediating VSTM storage. This conclusion rests on a single null result (chance-level decoding performance in V1–V4 during predictable distractor-present blocks in Experiment 1), and the results of subsequent experiments instead support the conclusion that both V1–V4 and sIPS contribute to VSTM storage, both in the presence (Experiment 3) and absence (Experiment 4) of distractors. These latter results agree with a growing body of evidence suggesting that VSTM is mediated by coordinated activity across multiple cortical regions. For example, recent studies have documented feature-specific VSTM representations in a multitude of visual, parietal, and prefrontal cortical areas (Sprague et al., 2014; Ester et al., 2015). Critically, some of these areas show classic signatures associated with VSTM, such as elevated delay period activation, but many others do not (Riggall and Postle, 2012; Ester et al., 2015). Moreover, artificial perturbations (eg, via transcranial magnetic stimulation) of neural populations within visual (van de Ven et al., 2012), parietal (Hamidi et al., 2008), and prefrontal (Fregni et al., 2005) cortex during VSTM alter memory performance, suggesting a functional role for each of these regions. Determining what role(s) these different regions play in mediating VSTM storage under different contexts is an important goal for future research. However, we suspect that even simple mnemonic behaviors, such as active storage of a single item over a short delay, depend on coordinated activity between a multitude of cortical areas, rather than just one or two “privileged” sites.

## References

[B1] Bettencourt KC, Xu Y (2016) Decoding the content of visual short-term memory under distraction in occipital and parietal areas. Nat Neurosci 19:150–157. 10.1038/nn.4174 26595654 PMC4696876

[B2] Christophel TB, Hebart MN, Haynes JD (2012) Decoding the contents of visual short-term memory from human visual and parietal cortex. J Neurosci 32:12983–12989. 10.1523/JNEUROSCI.0184-12.2012 22993415 PMC6621473

[B3] D’Esposito MD, Postle, BR (2015) The cognitive neuroscience of working memory. Annu Rev Psychol 66:115–142. 25251486 10.1146/annurev-psych-010814-015031 PMC4374359

[B4] Drew T, Vogel EK (2008) Neural measures of individual differences in selecting and tracking multiple moving objects. J Neurosci 28:4183–4191. 10.1523/JNEUROSCI.0556-08.2008 18417697 PMC2570324

[B5] Dubois J, de Berker AO, Tsao DY (2015) Single-unit recordings in the macaque face patch system reveal limitations of fMRI MVPA. J Neurosci 35:2791–2802. 10.1523/JNEUROSCI.4037-14.2015 25673866 PMC4323541

[B6] Emrich SM, Riggall AC, LaRocque JJ, Postle BR (2013) Distributed patterns of activity in sensory cortex reflect the precision of multiple items maintained in visual short-term memory. J Neurosci 33:6516–6523. 10.1523/JNEUROSCI.5732-12.2013 23575849 PMC3664518

[B7] Ester, EF, Serences, JT, Awh, E (2009) Spatially global representations in human primary visual cortex during working memory maintenance. J Neurosci 29:15258–15265. 10.1523/JNEUROSCI.4388-09.2009 19955378 PMC2830793

[B8] Ester EF, Anderson DE, Serences JT, Awh E (2013) A neural measure of precision in visual working memory. J Cogn Neurosci 25:754–761. 10.1162/jocn_a_00357 23469889 PMC4041615

[B9] Ester EF, Sprague TC, Serences JT (2015) Parietal and frontal cortex encode stimulus-specific mnemonic representations during visual working memory. Neuron 87:893–905. 10.1016/j.neuron.2015.07.013 26257053 PMC4545683

[B10] Fregni F, Boggio PS, Nitsche M. (2005) Anodal transcranial direct current stimulation of prefrontal cortex enhances working memory. Exp Brain Res 166:23–30. 10.1007/s00221-005-2334-6 15999258

[B11] Hamidi M, Tononi G, Postle BR (2008) Evaluating frontal and parietal contributions to spatial working memory with repetitive transcranial magnetic stimulation. Brain Res 1230:202–210. 10.1016/j.brainres.2008.07.008 18662678 PMC2612637

[B12] Harrison, SA, Tong F (2009) Decoding reveals the contents of visual working memory in early visual areas. Nature 458:632–635. 10.1038/nature07832 19225460 PMC2709809

[B13] Harvey BM, Klein BP, Petridou N, Dumoulin SO (2013) Topographic representation of numerosity in the human parietal cortex. Science 341:1123–1126. 10.1126/science.1239052 24009396

[B14] Mitchell DJ, Cusack R (2008) Flexible, capacity-limited activity of posterior parietal cortex in perceptual as well as visual short-term memory tasks. Cereb Cortex 18:1788–1798. 10.1093/cercor/bhm205 18042643

[B15] Nieder A, Diester I, Tudusciuc O (2006) Temporal and spatial enumeration processes in the primate parietal cortex. Science 313:1431–1435. 10.1126/science.1130308 16960005

[B16] Riggall AC, Postle BR (2012) The relationship between working memory storage and elevated activity as measured with functional magnetic resonance imaging. J Neurosci 32:12990–12998. 10.1523/JNEUROSCI.1892-12.2012 22993416 PMC3470886

[B17] Serences, JT, Ester EF, Vogel EK, Awh E (2009) Stimulus-specific delay activity in human primary visual cortex. Psychol Sci 20:207–214. 10.1111/j.1467-9280.2009.02276.x 19170936 PMC2875116

[B18] Sprague, TC, Ester EF, Serences JT (2014) Reconstructions of information in visual spatial working memory degrade with memory load. Curr Biol 24:2174–2180. 10.1016/j.cub.2014.07.066 25201683 PMC4181677

[B19] Todd, JJ, Marois, R (2004) Capacity limit of visual short-term memory in human posterior parietal cortex. Nature 428:751–754. 10.1038/nature02466 15085133

[B20] van Bergen RS, Ma WJ, Pratte MS, Jehee JFM (2015) Sensory uncertainty decoded from visual cortex predicts behavior. Nat Neurosci 18:1728–1730. 10.1038/nn.4150 26502262 PMC4670781

[B21] van de Ven V, Jacobs C, Sack AT (2012) Topographic contribution of early visual cortex to short term memory consolidation: a transcranial magnetic stimulation study. J Neurosci 32:4–11. 10.1523/JNEUROSCI.3261-11.2012 22219265 PMC6621312

[B22] Xu Y, Chun MM (2006) Dissociable neural mechanisms supporting short-term memory for objects. Nature 440:91–95. 10.1038/nature04262 16382240

